# Progress in CKD Care and Integration of Adult and Childhood Nephrology Services in Tanzania

**DOI:** 10.34067/KID.0000000000000477

**Published:** 2024-05-21

**Authors:** Francis F. Furia

**Affiliations:** School of Clinical Medicine and Muhimbili Renal and Rheumatology Research Group, Muhimbili University of Health and Allied Sciences (MUHAS), Dar es Salaam, Tanzania

**Keywords:** ESKD, hemodialysis, kidney transplantation

## Introduction

**Figure 1:**
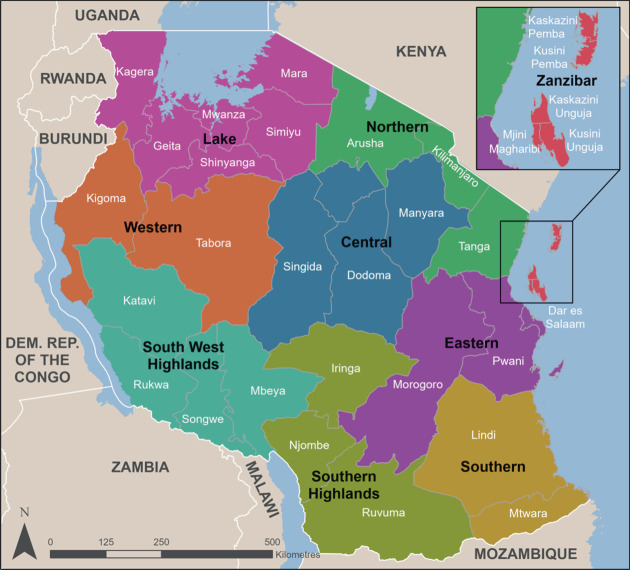
Map of Tanzania showing regions and zones.

Tanzania has a population of 61.7 million inhabitants; almost half (49%) of these are children aged 0–17 years. The country is geographically and administratively divided into 31 regions, each region has one regional referral hospital, and some regions are organized into zones served by zonal referral hospitals (Figure 1).

There are six zonal referral hospitals and one national hospital, Muhimbili National Hospital located in Dar es Salaam; five of the zonal hospitals are located in Kilimanjaro (Kilimanjaro Christian Medical Center), Mwanza (Bugando Medical Center), Dodoma (Benjamin Mkapa Hospital), and Mbeya (Mbeya Zonal Referral Hospital). The sixth zonal hospital, Mnazimmoja Hospital, is located in Zanzibar. Tanzania mainland and Zanzibar have separate ministries of health, and each ministry oversees services in their respective areas; however, Muhimbili National Hospital serves as the national hospital for both Tanzania mainland and Zanzibar.

Tanzania has a high burden of non-communicable diseases which is adding weight to the existing burden of infectious conditions including Malaria and HIV infections with a national prevalence of 4.4% as reported in the Tanzania HIV Impact Survey 2022–2023.^1^

Most Tanzanians are paying out of pocket for health services; 32% are reported to be using insurance, of which 26% are subscribed to the Community Health Fund, 8% to the National Health Insurance Fund (NHIF), and 1% subscribed to private insurance schemes. NHIF is a mandatory public scheme for public employees and offers comprehensive services, including reimbursement for RRT in the form of hemodialysis and immunosuppressive medications.

## Epidemiology of CKD in Tanzania

CKD is one of the predominant causes of morbidity and mortality in Tanzania, affecting both children and adults. The community-based prevalence of CKD among adults is reported to range between 7% and 14%,^2,3^ with CKD stage five prevalence of 3% from community-based study.^4^ The causes of CKD in Tanzania are presumed to be diabetes and hypertension; these two conditions have community prevalence rates of 6% and 28%, respectively.^5,6^

The burden of CKD among children is largely unknown. In one study that was conducted among children admitted to a tertiary hospital in North-western Tanzania, 16.2% of children were reported to have renal dysfunction; however, this report did not disaggregate the findings into acute kidney disease and CKD.^6^ Children with type 1 diabetes mellitus have been reported to have nephropathy, indicating a possible contribution of this condition to CKD among children.^7^

## CKD Services in Tanzania

### Hemodialysis

The number of hemodialysis units has increased dramatically in the past two decades. In 2006, there was only one hemodialysis unit in Tanzania; in 2015, there were 12 units, most of which were in big cities; this number rose to 28 units in 2019.^8^ The Ministry of Health established hemodialysis units in some of the regional referral hospitals and has plans for remaining regional referral hospitals. All dialysis units are offering maintenance therapy to patients, including those with HIV, hepatitis B, and hepatitis C viral infections. Currently, 47 hemodialysis units owned by public and private institutions provide services in Tanzania (Table 1).

**Table 1:** CKD services in Tanzania

Variable	*No.* or (%)
**Hemodialysis**	
No. of hemodialysis units	47
No. of hemodialysis machines	649
No. of patients on hemodialysis therapy	3231
No. of patients on home hemodialysis	0
The average duration of a dialysis session	4 h
No. of dialysis sessions per week	Three sessions for patients on NHIF, 1–2 sessions for patients paying out of pocket
Vascular access for hemodialysis therapy	All patients start hemodialysis on temporary access; approximately 40% have AVF the rest have catheters^9,10^
Cost of the hemodialysis session	USD 80–120
Dialysis funding for patients	>90% NHIF while the rest are covered by private insurances or paying out-of-pocket payment
Personnel who deliver hemodialysis therapy	Nurses
Ratio of patients: dialysis patients in dialysis units	8–10 patients to one nurse
**Peritoneal dialysis**	
Chronic peritoneal dialysis	0
**Kidney transplantation**	
No. of kidney transplant recipients to date	123
No. of kidney transplantation centers	2 (Muhimbili National Hospital and Benjamin Mkapa Hospital)
Induction immunosuppression medicines	Basiliximab, rituximab, and ATG-rabbit
Maintenance immunosuppression medicines	Prednisolone, tacrolimus, cyclosporine, mycophenolate mofetil/mycophenolic acid, azathioprine and everolimus

ATG, anti-thymocyte globulin; AVF, arteriovenous fistula; NHIF, National Health Insurance Fund.

### Peritoneal Dialysis

Peritoneal dialysis is offered in Tanzania, and this is mainly offered for patients with AKI.^8^ There is no established chronic peritoneal dialysis service in Tanzania, although few patients have managed to receive chronic peritoneal dialysis through individual patient/family initiatives.

### Kidney Transplantation

Kidney transplantation services in Tanzania were established in the 80s by sending donors and recipients out of the country, and the first batch was sent to the United Kingdom.^10^ Pretransplantation evaluations and post-transplantation care were provided in Tanzania at Muhimbili National Hospital. Muhimbili National Hospital started kidney transplantation services in 2017 and was followed by Benjamin Mkapa Hospital. A total of 123 patients have been transplanted to date in Tanzania.

The two hospitals offering kidney transplantation had collaborative partnerships with advanced centers that provided personnel training. Muhimbili National Hospital (MNH) collaborated with three Indian hospitals while Benjamin Mkapa Hospital collaborated with a Japanese institution.^10^ Training of the transplant teams for the two hospitals was conducted in India and Japan for MNH and Benjamin Mkapa Hospital, respectively.

Currently, only live-related kidney transplantation is offered in the two programs, and the immunosuppression medications used for induction include basiliximab, rituximab, and anti-thymocyte globulin-rabbit. Maintenance immunosuppression medications are predominantly prednisolone, tacrolimus, and mycophenolate mofetil/mycophenolic acid.

### Cost of RRT and Funding

Most patients receiving hemodialysis therapy are subscribers of the NHIF, which covers approximately 8% of the Tanzania population.^2^ NHIF reimburses the cost of dialysis therapy in both public and private dialysis units, and reimbursement is made for three sessions/week. The cost of a single hemodialysis session ranges from $80 to $120 while the cost of kidney transplantation surgery is approximately $12,000 with follow-up immunosuppression drugs cost of $240–$360 per month. The Ministry of Health is currently engaging different stakeholders, including the Nephrology Society of Tanzania, to find strategies for reducing these costs which are not affordable.

## Integration of Adult and Pediatric CKD Care in Tanzania

The Muhimbili University of Health and Allied Sciences established nephrology training in Tanzania in 2007 through collaboration with the University of Bergen, Norway, and Christian Medical College, Vellore, from 2007 to 2014.^10^ The program is hosted in the Department of Internal Medicine and admits internal medicine and pediatric physicians. A total of 23 nephrologists have been trained, of which eight are pediatricians.

Nephrology services at Muhimbili National Hospital are offered in one setting for adults and children. This has been made possible by the training model that allows pediatricians and internal medicine physicians to train in a single nephrology program that is offered at Muhimbili University of Health and Allied Sciences. The nephrology unit at MNH has nephrologists with pediatric and internal medicine backgrounds, and they both see patients in one clinic and one hemodialysis unit. They also attend to admitted patients in general medical wards, pediatric wards, intensive care, and pediatric intensive care units.

Nephrology trainees are also trained in the same setting, making it possible for them to manage common adult and childhood nephrology conditions regardless of their backgrounds in pediatrics or internal medicine. These nephrologists will provide comprehensive nephrology services to adults and children in their facilities once they graduate.

## Conclusions

Tanzania has a significant burden of CKD, and like other countries in resource-limited settings, there is a challenge of health financing, with most of the people paying out of pocket. Significant advances have been made in providing CKD services with the expansion of hemodialysis services and the establishment of kidney transplantation services. More efforts are required to strengthen existing services and introduce peritoneal dialysis services.
